# Dynamic *in-situ* sensing of fluid-dispersed 2D materials integrated on microfluidic Si chip

**DOI:** 10.1038/srep42120

**Published:** 2017-02-10

**Authors:** Benjamin T. Hogan, Sergey A. Dyakov, Lorcan J. Brennan, Salma Younesy, Tatiana S. Perova, Yurii K. Gun’ko, Monica F. Craciun, Anna Baldycheva

**Affiliations:** 1 Department of Engineering and Centre for Graphene Science, College of Engineering, Mathematics and Physical Sciences, University of Exeter, Exeter, EX4 4QF, UK.; 2 EPSRC Centre for Doctoral Training in Electromagnetic Metamaterials, University of Exeter, EX4 4QL, UK.; 3 Skolkovo Institute of Science and Technology, Photonics and Quantum Materials Centre, Nobel Street 3, Moscow, Russia; 4 School of Chemistry and CRANN, Trinity College Dublin, the University of Dublin, Dublin 2, Ireland; 5 École Nationale Supérieure de Mécanique et des Microtechniques, Besançon, France; 6 Department of Electronic and Electrical Engineering, Trinity College Dublin, the University of Dublin, Dublin 2, Ireland; 7 ITMO University, 49 Kronverkskiy pr., St.-Petersburg, 197101, Russia

## Abstract

In this work, we propose a novel approach for wafer-scale integration of 2D materials on CMOS photonic chip utilising methods of synthetic chemistry and microfluidics technology. We have successfully demonstrated that this approach can be used for integration of any fluid-dispersed 2D nano-objects on silicon-on-insulator photonics platform. We demonstrate for the first time that the design of an optofluidic waveguide system can be optimised to enable simultaneous *in-situ* Raman spectroscopy monitoring of 2D dispersed flakes during the device operation. Moreover, for the first time, we have successfully demonstrated the possibility of label-free 2D flake detection via selective enhancement of the Stokes Raman signal at specific wavelengths. We discovered an ultra-high signal sensitivity to the *xyz* alignment of 2D flakes within the optofluidic waveguide. This in turn enables precise *in-situ* alignment detection, for the first practicable realisation of 3D photonic microstructure shaping based on 2D-fluid composites and CMOS photonics platform, while also representing a useful technological tool for the control of liquid phase deposition of 2D materials.

The recent development of nanocomposites consisting of fluid-dispersed atomically thin two-dimensional (2D) materials has sparked a great level of interest as a highly promising *in-situ* tailored meta-material device platform for the next generation of multi-functional optoelectronic systems^1^,^2^,^3^,^4^,^5^,^6^,^7^,^8^,^9^,^10^,^11^,^12^,^13^. Dynamically controlled three-dimensional (3D) self-assembly of fluid-suspended 2D materials not only provides a new breakthrough route for technological applications of 2D material based 3D device architectures^14^,^15^,^16^, but also their fluidic nature allows CMOS-compatible wafer-scale back-end integration on chip using microfluidic technology^17^,^18^,^19^. The practical realisation of dynamically reconfigurable 3D material fluid metastructures integrated into a microfluidic system and coupled with a CMOS photonic circuit (as illustrated in Fig. 1) opens up limitless possibilities in the fabrication of compact and low-power systems for commercially viable, miniaturised, multi-functional light-management devices such as light emitting sources^20^, tuneable optical filters^21^ and nanoantenna phased arrays^22^. For example, 1D photonic crystals of graphene which could act as optical filters have been theoretically explored^23^ but as yet no method exists for the precise reconfigurable control of such structures. Additionally, self-assembly of fluid dispersed 2D materials represents a remarkable precursor state for liquid phase deposition of ordered lamellae, stacks and other structures in the solid phase^24^,^25^,^26^. Reconfigurability of the metastructures can be achieved by exploiting liquid crystal technology^6^,^15^ with *in-situ* monitoring used to characterise the metastructure formation.

However, the primary challenge for the first realisation of on-chip controlled assembly of 2D flakes into functional metastructures is the lack of a reliable and sensitive method for *in-situ* characterisation. Such a method is pivotal to the confirmation of the integration of composites in the device and to enable determination of parameters, such as spatial alignment of incorporated 2D materials, dynamically during device operation. Conventional characterisation methods such as Raman spectroscopy^5^,^27^,^28^, coherent anti-Stokes Raman spectroscopy (CARS)^29^ and Fourier Transform Infrared Spectroscopy (FTIR)^30^ are not suitable for studies of fluid nanocomposites with relatively low concentrations of nanoparticles dispersed. In this case, the significantly greater scattering volume of the host fluid compared to that of the dispersed nanoparticles always dominates the vibrational signal intensity, rendering the monitoring of the considerably weaker bands of dispersed nanoparticles impossible.

Here we demonstrate, for the first time, the wafer-scale integration of fluid-dispersed 2D materials on Si photonic chip utilising microfluidic technology, and their subsequent electrical and optical manipulation. We also propose and subsequently demonstrate a novel approach for ultra-sensitive, label-free, *in-situ* detection and monitoring of integrated 2D-fluid composite materials on-chip. Specifically, we propose an *in-situ* micro-Raman characterisation approach, whereby the Raman signal of 2D dispersed nanoparticles is selectively enhanced through the design of optofluidic waveguide geometry on silicon-on-insulator (SOI) platform. It has previously been shown that the Raman signal from micro-structured silicon cavities can be enhanced due to Fabry-Pérot type resonances^31^,^32^,^33^. Here, the structure is designed to simultaneously enhance different resonant modes relating to different parameters of the optofluidic waveguides, balancing the required enhancement of the signal from multiple vibrational bands with the desire for the greatest achievable intensity for the individual bands. The developed approach demonstrates ultra-high sensitivity to the *xyz* alignment of 2D nanoparticles within optofluidic waveguides. Hence, for the first time, our findings demonstrate the possibility of monitoring the dynamics of fluid-dispersed 2D nanoparticles on chip. Our work paves the way for the practical realisation of dynamically reconfigurable photonic metastructures based on 2D-fluid composites integrated on CMOS photonics platform with a range of important applications, such as renewable energy, optical communications, bio-chemical sensing, and security and defence technologies^4^,^8^,^34^ or as a precursor to controlled deposition of solid state structures^24^,^25^,^26^.

Typically, large-scale CMOS photonics builds on a SOI platform; a high index-contrast waveguide platform which prevents interference between the photonic integrated circuit components and the substrate. Therefore, we use an SOI based Fabry-Pérot type optofluidic waveguide channel, with an open top cladding (Fig. 1 inset) to allow *in-situ* micro-Raman detection and monitoring of the integrated 2D fluid nanocomposite system during device operation. To optimise the optofluidic waveguide design for facilitating strong confinement of light on chip and to significantly enhance the Raman back-scattered signal of the individual incorporated 2D nanoplatelets, we model the variation in the intensity of the Raman bands of dispersed nanoparticles while varying parameters that can be experimentally controlled, such as: the waveguide width, *w*, and the buffer oxide (BOX) layer thickness, *h*_*BOX*_. We consider the specific case of the D and G bands of 2D carbon-based materials (Fig. 2)- such as graphene and graphene oxide (GO)- dispersed in a nematic liquid crystal (LC) host, however the proposed methodology can be utilised for any fluid-dispersed material. The backscattered Raman signal intensity is numerically determined for wavelengths corresponding to the Raman active bands using the scattering matrix method [See Supplementary Methods]. The complex variation of the Raman signal as a result of modifying the optofluidic waveguide parameters can be rationalised as the superposition of Fabry-Pérot resonance effects in different parts of the geometry. Enhancement of up to 100x can be observed between maximising and minimising combinations of the optofluidic waveguide parameters.

In order to experimentally demonstrate the enhancement of the Raman intensity, 2D material-fluid nanocomposites, consisting of graphene and GO nanoplatelets dispersed in LCs, were prepared by a liquid phase dispersion method [See Supplementary Methods]. The optimal parameters for the microfluidic structures were determined from the calculated Raman signal intensity maps shown in Fig. 2. Resonator devices consisting of optofluidic waveguide channels of different widths were fabricated on SOI wafer with a thick buffer oxide (*h*_*BOX*_ = 2 *μm*) layer and with a silicon device layer of 15 μm. The prepared nanocomposites were integrated into optofluidic waveguide channels via infiltration reservoirs on the chip (Fig. 3). The selected microfluidic channels had widths in the ranges 3.7 ± 0.2 *μm* (narrow channels, strong enhancement) and 10.5 ± 1.5 *μm* (wide channels, weaker enhancement). Optical microscopy (Fig. 3b) and scanning electron microscopy (Fig. 3c–e) both confirmed the successful integration of the nanocomposite into all channels.

Raman spectra are presented for the GO-LC nanocomposites- with MLC-6608 (Fig. 4a) and with E7 (Fig. 4b) as the fluid host-measured *in-situ* at three points on the chip; more specifically: in a wide channel (Fig. 4c), in a narrow channel (Fig. 4d) and in an infiltration reservoir (Fig. 4e). Liquid crystal MLC-6608 exhibits weak Raman bands (see Supplementary Results) that have no strong overlap with the D and G bands, allowing for clear determination of the GO bands in gathered spectra. Raman spectra were recorded for individual monolayer flakes of area 1.0 ± 0.1 *μm*^*2*^ in all cases. For GO dispersed in MLC-6608, the D band Raman intensities were observed in the approximate ratio 5:8:16 for an infiltration reservoir, 11.6 *μm* channel and 3.6 *μm* channel respectively. For the G band, intensities were observed in the ratio 5:7:16. E7, however, has a strong Raman active vibrational band at around 1605 *cm*^*−1*^ (see Supplementary Results), overlapping with the G band. Nevertheless, utilising the proposed signal enhancing design, the observation of the G band as a broad shoulder on this band is feasible (Fig. 4b). In addition, similar results were obtained for nanocomposites with graphene instead of GO.

The close agreement, in Fig. 4f, between the relative intensities of the D and G bands found experimentally for all nanocomposites (points) and those determined numerically (solid lines) verifies the method for predicting the Raman signal enhancement. For both the D and G bands, the numerically determined enhancement ratio was within the error of the experimental measurements; slight differences occur due to the flake not being positioned precisely at the centre of the channel. Therefore, this technique presents an effective tool for maximising the enhancement of the Raman signal.

Understanding the dynamics of 2D nanoplatelet spatial alignment is essential for the realisation of three-dimensional metastructure formation. External stimuli, such as applied electric field and light coupling^6^,^15^ [See Supplementary Results and Supplementary Videos 1,2,3,4], induce dynamic re-ordering of suspended 2D nanoparticles. Here, a Raman laser was exploited to move a GO flake within a channel (Fig. 5a), while simultaneously being used to monitor the *xyz* alignment over time. The variation in the experimental Raman spectra for different flake positions within the channel is illustrated in Fig. 5b.

The effect of the flake position on the Raman signal intensity was modelled by varying the position of the oscillating dipoles within the optofluidic waveguide channel both laterally and vertically. The ratio of the D and G band intensities is not constant as the position is varied (Fig. 5c–d). For lateral displacements (Fig. 5c), there are ratios of 11 and 32 between the minimal and maximal intensities determined numerically for the D and G bands respectively. For vertical displacements (Fig. 5d), the maximum and minimum values of the average intensity differ by factors of around four and five times for the D and G bands respectively.

For the lateral displacement of the flake, the ratios of the intensities of the GO D and G bands were extracted from experimental spectra when a flake was next to the wall and when moved further towards the centre of the channel. These ratios were then used as a multiplier on the numerically determined intensity for the flake next to the wall (position 2 in Fig. 5a) to determine an approximate displacement. Positions approximated using experimental data in tandem with the numerically determined results are closely matched to the values observed using optical microscopy techniques (See Supplementary Results), falling within the experimental error. The lateral position can therefore be determined with similar precision to optical microscopy (approx. ± 5%) currently, but with scope for the error to be reduced significantly by improving the signal-to-noise ratio.

For the vertical displacement of the flake, again the ratios of the intensities of the GO D and G bands were extracted, this time from data with the flake at the bottom of the channel and further towards the surface. The ratios were then used to multiply the numerically determined intensity with the flake at the bottom of the channel to determine an approximate displacement. The numerical analysis covering the effect of vertical flake position shows close agreement with the experimental data. The vertical position of the flake cannot be determined from optical microscopy but the close agreement of the positions determined separately from the D and G bands confirms that the method is accurate. Therefore, the predictions made from the Raman spectra are the most accurate method of determining the vertical position currently available. While we propose this technique as a method for monitoring self-assembly of 3D metastructures comprised of 2D materials, this approach may also find applications in a wide range of other areas such as controlling flake alignment for liquid phase deposition of 2D materials^24^,^26^ or for spatial monitoring of nanoparticle distributions^35^.

In summary, we propose a novel approach for integration of 2D materials on CMOS photonic chip utilising microfluidics technology. We have successfully demonstrated that this approach can be used for integration of any fluid-dispersed 2D nanoparticles on SOI photonics platform. The optofluidic system design can be optimised to enable *in-situ* Raman spectroscopy monitoring of 2D dispersed flakes during device operation. *In-situ* label-free 2D flake sensing via selective enhancement of the Stokes Raman signal at given wavelengths has been determined numerically and confirmed experimentally. This approach has been applied to monitor the individual 2D nanoplatelet dynamic within an optofluidic waveguide with high sensitivity, enabling precise *in-situ* alignment monitoring for the first practical realisation of 3D photonic metastructure shaping based on 2D-fluid composites and CMOS photonics platform.

## Additional Information

**How to cite this article**: Hogan, B. T. *et al*. Dynamic *in-situ* sensing of fluid-dispersed 2D materials integrated on microfluidic Si chip. *Sci. Rep.* **7**, 42120; doi: 10.1038/srep42120 (2017).

**Publisher's note:** Springer Nature remains neutral with regard to jurisdictional claims in published maps and institutional affiliations.

## Supplementary Material

Supplementary video 1

Supplementary video 2

Supplementary video 3

Supplementary video 4

Supplementary Information

## Figures and Tables

**Figure 1:**
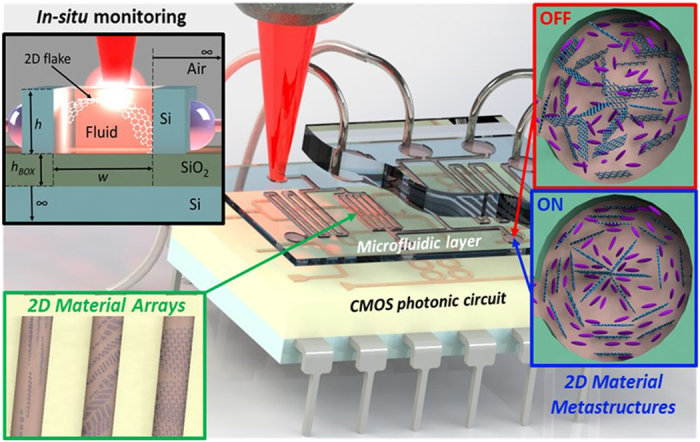
A CMOS photonic circuit coupled to a microfluidic layer integrating dynamically reconfigurable 2D material metastructures by exploiting liquid crystal technology. *In-situ* micro-Raman spectroscopy detection and monitoring gives information on the formation of the metastructures.

**Figure 2:**
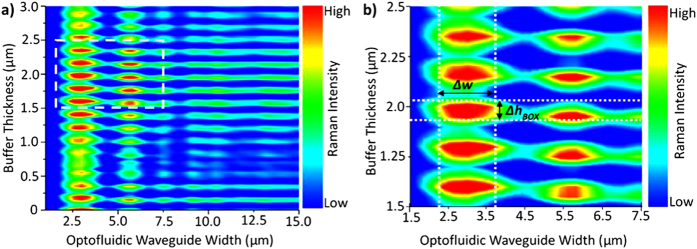
(**a**) Map showing regions where both the D and G bands of 2D carbon-based materials are determined numerically to be strongly enhanced in Raman spectroscopy measurements. (**b**) Expanded view of the region of interest highlighted in a. The range of parameters for which strong enhancement is observed are shown with the white lines including fabrication deviation *Δh*_*BOX*_ and *Δw*.

**Figure 3:**
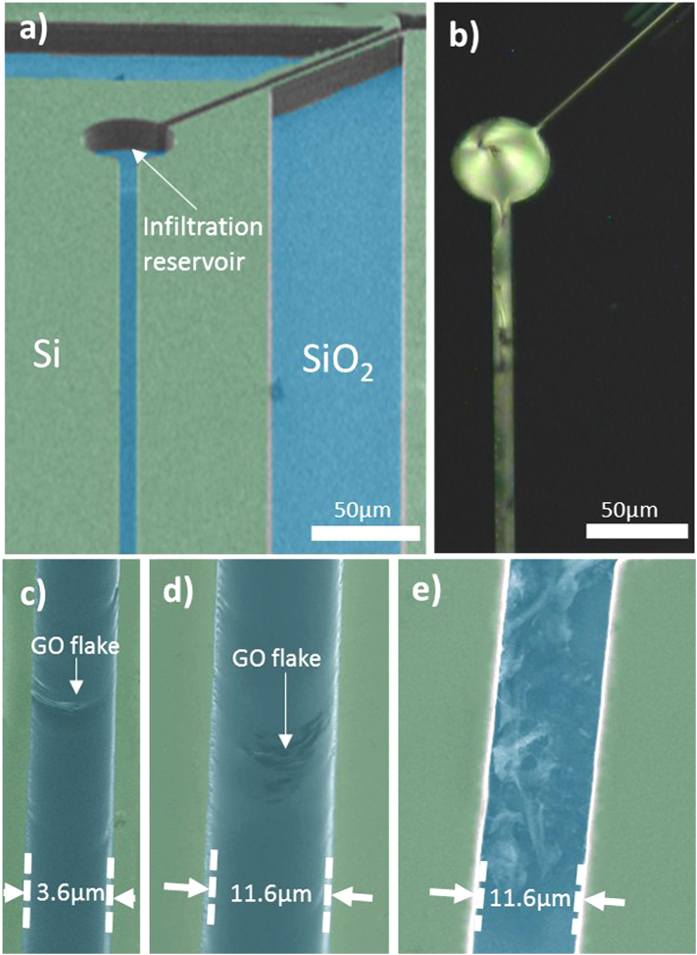
(**a**) SEM image of the chip used for Raman measurements, before infiltration with the nanocomposite. (**b**) Polarised microscopy image of the structure infiltrated with a composite of MLC 6608 and graphene oxide. Integration of the composite, including GO flakes, into all microfluidic structures on the chip can be seen. (**c**) SEM of GO flakes infiltrated with a host LC into a 3.6 μm channel. (**d**) SEM of GO flakes infiltrated with a host LC into an 11.6 μm channel. (**e**) SEM of an 11.6 μm channel, with the LC removed, showing the integration of large numbers of GO flakes.

**Figure 4:**
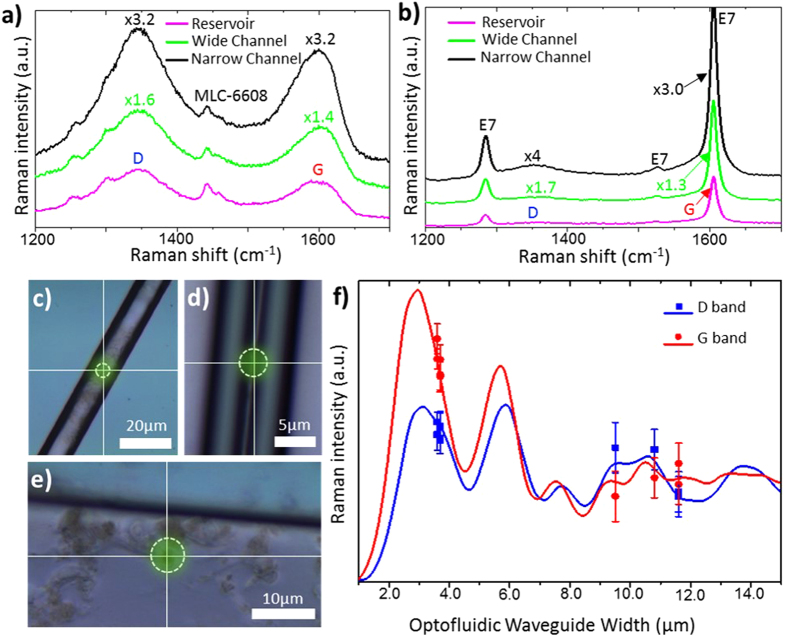
(**a,b**) Normalized Raman spectra showing the enhancement of the D and G bands for graphene oxide dispersed in (**a**) liquid crystal MLC 6608 and (**b**) liquid crystal E7. Spectra are shown for three microfluidic geometries: in (**c**) an infiltration reservoir of width 100 μm (magenta), in (**d**) a microfluidic cavity of width 11.6 μm (green) and in (**e**) amicrofluidic cavity of width 3.6 μm (black). Approximate laser spot sizes are shown in (**c–e**). (**f**) Comparison of numerically determined (solid lines) and experimentally measured (points) Raman intensities of the graphene oxide D (blue) and G (red) bands. All data is normalized to the case where the walls are separated by a distance great enough for Fabry-Pérot resonances to have no effect.

**Figure 5:**
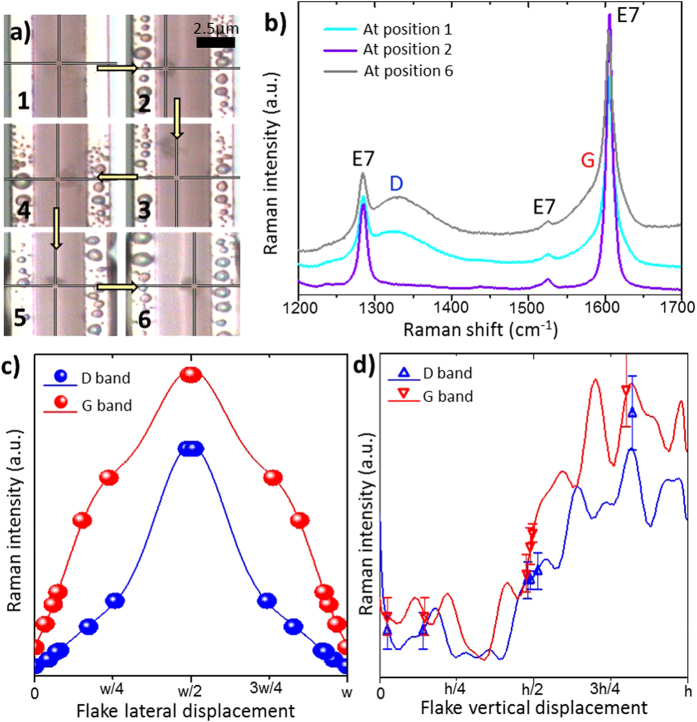
(**a**) GO flake movement induced by the Raman laser. Each image represents the change after 10 *s* exposure time in the order in which they were observed. (**b**) Raman spectra of a GO flake dispersed in liquid crystal E7 within a narrow channel (approx. 3.6 *μm*) at positions 1 (cyan), 2 (violet) and 5 (grey) as seen in **a**. (**c–d**) The variation of the Raman intensity of the GO D (blue) and G (red) bands for lateral (**c**) and vertical (**d**) displacements of a GO flake within the microfluidic channel. Solid lines give the numerically determined Raman intensities. Flake positions determined from normalized experimental spectra are shown as points. For lateral displacements, the error in the experimental measurement is given by the size of the symbols.
