# Supplementary material for: Dynamic in-situ sensing of fluid-dispersed 2D materials integrated on microfluidic Si chip
Source: Sci Rep. 2017 Feb 10;7:42120. doi: 10.1038/srep42120 (PMC5301493; doi:10.1038/srep42120)
Supplement: Supplementary Information [file srep42120-s5.pdf]

Dynamic *in-situ* sensing of fluid-dispersed 2D materials integrated on microfluidic Si chip

Benjamin T. Hogan, Sergey A. Dyakov, Lorcan J. Brennan, Salma Younesy, Tatiana S. Perova, Yurii K. Gun'ko, Monica F. Craciun and Anna Baldycheva

Supplementary Methods

Numerical determination of the Raman signal intensity using the scattering matrix method. The scattering matrix method (SMM) is a powerful tool for numerical determination of the near- and far-field light distribution for structures which can be split into layers uniform along at least one direction[1–4]. The main principle of this method is the decomposition of electric and magnetic fields into Fourier series in each layer and connecting the Fourier components of adjacent layers in accordance with the boundary conditions of Maxwell's equations.

In this work, we propose an SOI based optofluidic waveguide channel design (Fig. 1 inset) for in-situ micro-Raman detection and monitoring of integrated 2D fluid nanocomposite. We optimised the optofluidic waveguide design to significantly enhance the back-scattered Raman signal from incorporated 2D nanoplatelets. Raman scattering is a quantum mechanical process with a random spatial distribution of the photons involved, however the optical behaviour of the scattered light can be modelled using a classical electrodynamics approach. 2D flakes within nanocomposites are modelled as a system of chaotically-oriented oscillating electrical dipoles[5] within a microfluidic channel, with the dipole emission defining the Raman signal wavelength. To numerically determine the far-field intensity of the dipole emission the SMM was used.

To reach convergence, 801 Fourier harmonics were used. The local components of the electromagnetic field were found, forming material matrices in each layer. By applying an iterative

procedure, the total scattering matrix for the whole structure was calculated[6]. The back-scattered Raman intensity was calculated from the components of the scattering matrix.

All numerical analyses were made for normal angles of Raman laser incidence and signal collection. The electric field vector of the incident light is oriented parallel to the channel walls. The nematic LC host is a birefringent material, however, an advantage of the microfluidic infiltration into SOI cavities is the spontaneously induced planar alignment of the LC through interaction with the surfaces of the Si walls[7]. The LC will therefore have a director which is either parallel or perpendicular to the walls of the channel such that only either the extraordinary or ordinary refractive index is required.

The spot size of the Raman laser was effectively considered as equal to the microfluidic cavity width. To account for this, all Raman intensities were normalised by accounting for the incident field strength in the channel. Since the incident and Raman-scattered wavelengths of the light are considerably greater than the thickness of 2D flakes in nanocomposites, the flake is considered to be a point dipole- an emitter of only Stokes or anti-Stokes photons within the system- and hence the refractive index of the flake material has no effect on the propagation of backscattered light in the cavity. For Fabry-Pérot effects in a layer to be observed experimentally, its thickness should be less than the coherence length of the Raman scattered light[5]. However, this condition is not fulfilled for the silicon substrate layer, hence it is modelled as a semi-infinite material by removing all Fabry-Pérot resonances within the substrate layer. Very thick silicon walls were considered; the thickness of the silicon walls has been shown to have little effect on the intensities determined by numerical analysis.

Numerical determination of the effect of flake position on the Raman signal intensity. The effect of the flake position on the Raman signal intensity was modelled, using the SMM, by varying the position of the oscillating dipoles within the optofluidic waveguide channel both laterally and vertically. Vertical displacements were defined from the bottom surface of the channel and lateral displacements from a side wall. For vertical displacement variation, the flake was fixed at the lateral

centre while for lateral displacement variation it was fixed at the vertical centre. The liquid crystal in this case was defined as commercially available E7. We chose parameters of the optofluidic waveguide that demonstrate strong enhancement of both the D and G bands.

Calculation of LC refractive indices. The ordinary and extraordinary refractive indices were calculated at all wavelengths subsequently utilised in the numerical analysis by utilising a three-coefficient Cauchy model, $n_{o,e} = A_{o,e} + \frac{B_{o,e}}{\lambda^2} + \frac{C_{o,e}}{\lambda^4}$; where n is the refractive index and A, B and C are suitable fitting parameters. The subscripts o and e represent the ordinary and extraordinary indices respectively. For example, for commercially available LC MLC 6608, A_o, B_o and C_o were set to 1.4609, $5\mathrm{x}10^{-3}$ μm^2 and 0 μm^4 respectively to give good agreement with experimental measurements in the visible region[8]. As MLC 6608 has a low birefringence in the visible region, the two-coefficient Cauchy model is a sufficient approximation.

Synthesis of GO-LC nanocomposites. Graphene oxide (GO) was prepared from bulk graphite *via* the Hummers method[9]. 1 *g* of graphite, 0.5 *g* of sodium nitrate and 23 *mL* of sulfuric acid (H_2SO_4) were added to a 500 *mL* round bottomed flask contained within an ice bath and stirred at 4 *°C* for 15 minutes. 3 *g* of potassium permanganate ($KMnO_4$) was added slowly with vigorous stirring. Once all the $KMnO_4$ was added, the ice bath was removed and the suspension was heated to 35 *°C* for 30 minutes. This produced a murky brownish-grey solution. Following this, 46 *mL* of water was added and the suspension was set to stir for 15 minutes. The solution was then treated with 1.4 *mL* of hydrogen peroxide (H_2O_2). The product was washed through centrifugation up to 10 times with a 10 % aqueous solution of hydrochloric acid (HCl) followed by copious amounts of deionised water. The resulting GO was suspended in H_2O and filtered under vacuum onto an omniporous 200 *nm* membrane, washed with 1 *L* of H_2O and then dried at 80 *°C* for several days. Once dried, the GO was dispersed in tetrahydrofuran (THF) through ultrasonication, exfoliating the material down to a few layers[10]. The dispersions were then centrifuged, allowing for extraction of the lowest mass GO flakes from the top layer of the suspension. An aliquot from this layer was mixed with the liquid crystal and

the mixture underwent further ultrasonication to ensure thorough mixing of the two components. The resulting dispersion was dried in a Schlenk flask, under vacuum, allowing for complete evaporation of the residual solvent, resulting in a nanocomposite of GO nanoplatelets uniformly dispersed in the liquid crystal. The concentration of GO in the final nanocomposites was approximately 0.01 $g.ml^{-1}$. Dispersed flakes had average sizes of around 1 μm^2 and were typically found to be between 1 and 5 layers thick. The number density of graphene oxide particles was calculated to be approximately 10^{12} *particles/ mL*. Graphene nanocomposites were produced by a similar method starting from bulk graphite powder.

Instrumentation. Scanning electron microscopy (SEM) measurements were performed on a Hitachi S3200N system with a practical operational magnification between 20-60000x, accelerating voltages from 0.3 to 30 *kV*, vacuum chamber pressure <0.1 *mbar* and a maximum resolution of 3.5 *nm*. Micro-Raman measurements were performed using a Renishaw 1000 system (with a 514.5 nm excitation wavelength from Ar^+ laser, a power of 5 *mW* and a spot size of approximately 3 *μm*) and on a Horiba Raman system (with an excitation wavelength of 532 *nm*, a power of 8.75 *mW* and a spot size of approximately 5 *μm* when focused through a x50 objective). Polarised light images were obtained using a Zeiss Axioscope 2 microscope with a Zeiss Axiocam MRc 5 camera, with x20 and x50 objectives used.

Supplementary Results

Raman intensity maps. Maps were produced separately for the D and G bands of 2D carbon-based materials such as graphene or GO (Sup. Fig. 1). Strong variations in the Raman intensity are observed as various parameters of the optofluidic waveguide are varied.

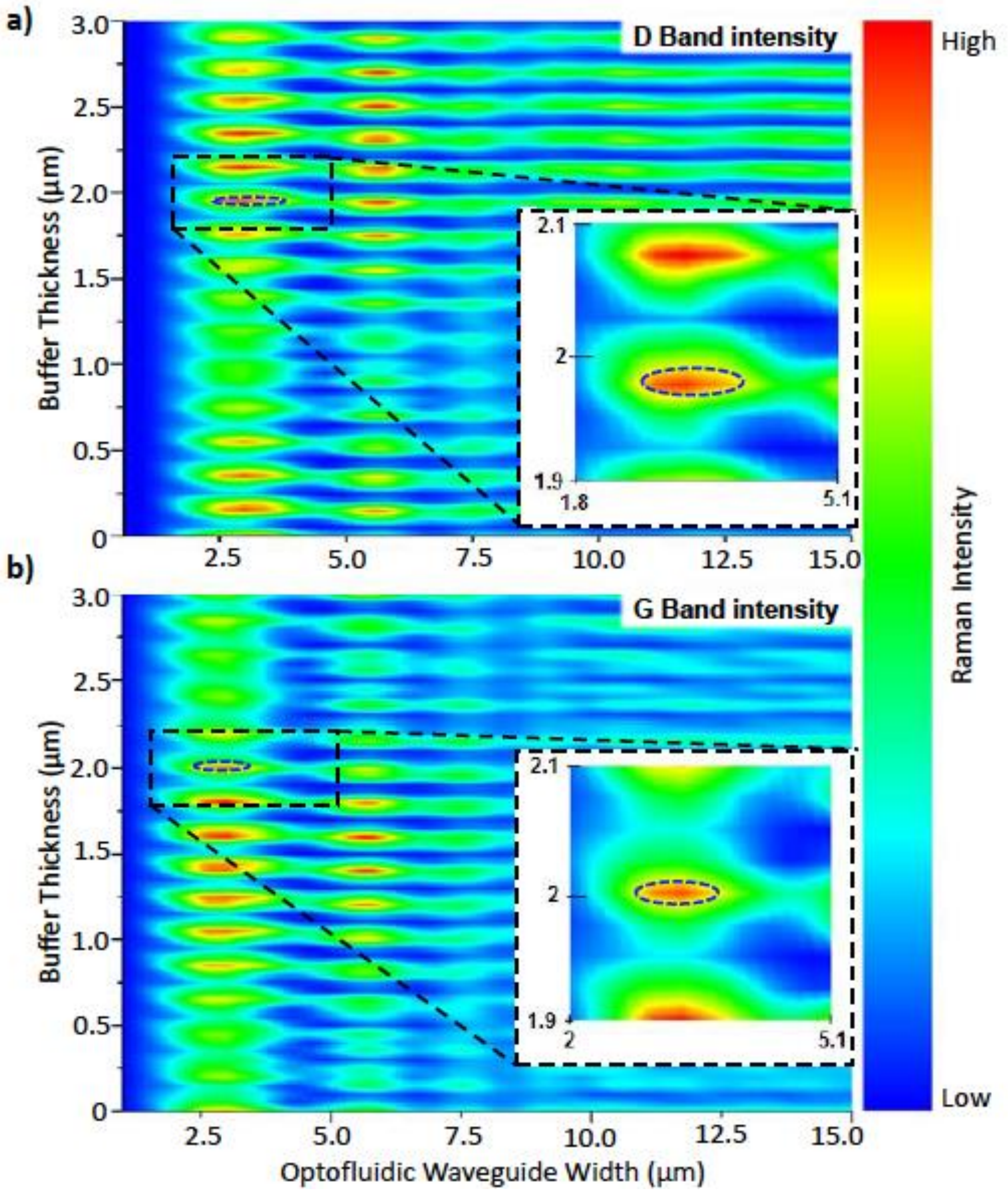

Supplementary figure 1: Maps of the Raman signal intensity at wavelengths corresponding to **a**) the D band and **b**) the G band of fluid-dispersed 2D carbon-based materials, under excitation by a 532nm Raman laser, as the optofluidic waveguide width and buffer oxide layer thicknesses are varied. High intensity areas of interest are highlighted.

Liquid crystal spectra. The liquid crystal host materials used for the nanocomposites in experimental measurements have several characteristic bands in their Raman spectra (Sup. Fig. 2) which are then also present in the spectra of the nanocomposites. The spectra were recorded for droplets of the liquid crystal greater than 100 μm deep, on a SOI substrate. The spectrum for MLC 6608 has been scaled by a factor of four. E7 shows a strong Raman active vibrational band at around 1605 cm^{-1}, overlapping with the G band of graphene oxide. There is an additional strong band at around 1280 cm^{-1}, which partially overlaps with the D band of graphene oxide, as well as two very weak bands at around 1440 cm^{-1} and 1525 cm^{-1}. MLC 6608, however, only shows weak bands at around 1245 cm^{-1}, 1305 cm^{-1}, 1355 cm^{-1}, 1450 cm^{-1}, 1460 cm^{-1} and 1640 cm^{-1}.

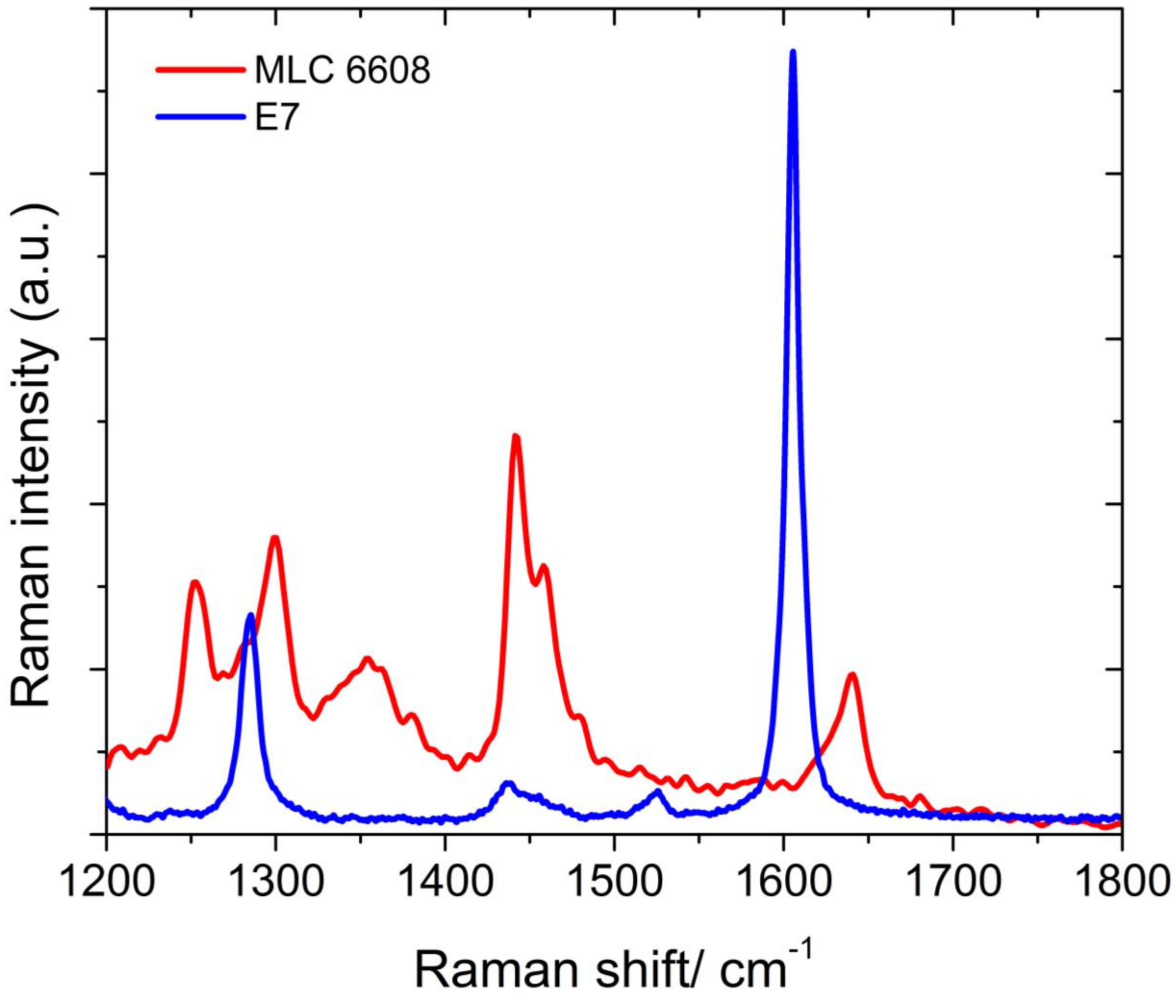

Supplementary figure 2: Raman spectra for droplets of the liquid crystal host fluids used in the synthesised nanocomposites on a SOI substrate: MLC 6608 (red) and E7 (blue). The spectrum for MLC 6608 has been scaled by a factor of four.

Nanoparticle alignment by external stimuli. The GO flakes dispersed in the LC can be manipulated by re-orienting the LC director (See Sup. Vid. 1). Applying an electric field across the channel results in a switching of the orientation of the LC molecules when a threshold voltage between the two walls is reached[11,12] (See Sup. Vids. 2-3). This is observed as a change from a bright to a dark state in polarised optical microscopy. For the GO dispersion in LC E7, an off-chip threshold voltage of approximately 3.5 *V* was observed in a 50 *μm* wide channel (Sup. Fig. 3). As the voltage was increased further above the threshold voltage, bands of light and dark states appeared, with the light state bands centred around GO flakes positioned next to the channel walls. Smaller flakes in the centre of the channel are observed to move as the voltage is increased.

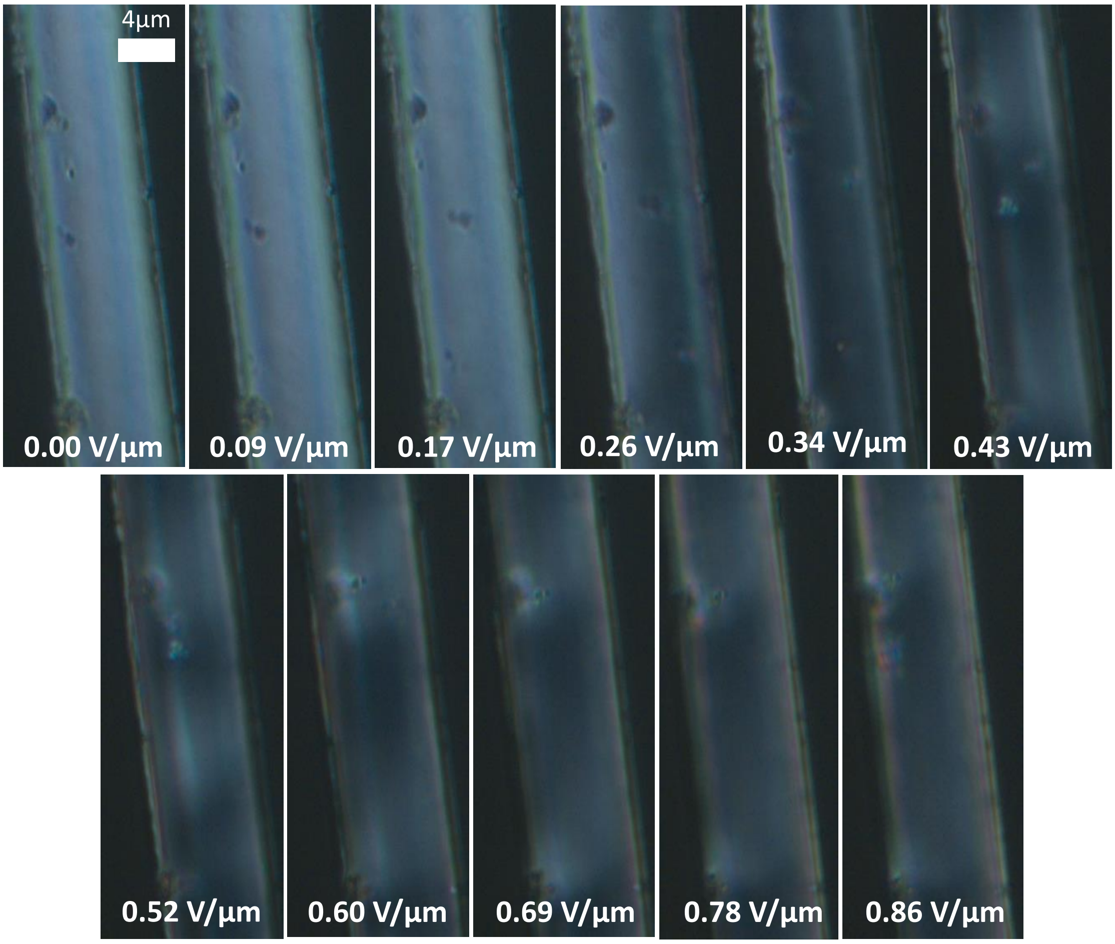

Supplementary figure 3: Polarized optical microscopy images showing the change in orientation of liquid crystal E7, with GO flakes dispersed, as the applied field strength between the walls of an 11.6 *μm* wide channel is increased.

The Raman laser can also be used to induce repositioning and reorientation of the dispersed flakes[13]. Flakes can be induced to rise within the fluid host or to move towards (or away from) the walls of the channel controllably. Flake movement can be observed as strong changes in the

interference pattern observed in the back-scattered light. In particular, flakes were shown to be drawn towards the laser spot and to circle the centre of the spot (See Sup. Vid. 4) when the laser intensity was suitably high. This allowed controllable repositioning of the flakes within the microfluidic channels.

In-situ *detection of nanoparticle positions.* The positions of the individual GO flakes within the liquid crystal host can be accurately predicted from the Raman spectra, specifically by looking at the G and D band intensities relative to a reference position. Supplementary Table 1 summarises the predicted positions shown in Fig. 5c of the main text. For each of the six experimental positions determined, there was a strong agreement between the predictions made using the D and G band intensities respectively, with the greatest disparity being just 2 *%* of the optofluidic waveguide width.

Supplementary table 1: Summary of positions predicted for various flake alignments by comparing to reference data (*) normalised relative to numerically determined values.

Flake designation	Normalised D band intensity (a.u.)	Position corresponding to D band intensity (% of cavity width)	Normalised G band intensity (a.u.)	Position corresponding to G band intensity (% of cavity width)	Difference between D and G positions (*%* of cavity width)
Ref*	0.05*	0.000<br>1.000	0.18*	0.000<br>1.000	0.0000
A	0.09597	0.045<br>0.955	0.33937	0.034<br>0.966	1.1000
B	0.14839	0.071<br>0.929	0.47437	0.060<br>0.940	1.1000
C	0.15806	0.081<br>0.920	0.56063	0.075<br>0.925	0.5500
D	0.32177	0.173<br>0.827	1.05563	0.153<br>0.847	2.0000
E	0.5	0.258<br>0.742	1.35	0.238<br>0.762	1.9230
F	1.55	0.489<br>0.511	2.06	0.496<br>0.504	0.7000

For each experimental flake position, there are two predicted values given for the D and G peaks respectively. This is due to centrosymmetric nature of the cavity in the *x* direction. By breaking this symmetry- for example by using an off-centre laser spot or by having walls of different permittivity-

the numerical analysis would no longer return a centrosymmetric intensity profile which may lead to a single position being determinable.

For flake position A, optical microscopy determined that the flake was aligned precisely next to the wall of the optofluidic waveguide. The position as determined from the Raman spectrum suggested the flake was displaced by around 4.5 % if looking at the D band or 3.4 % if looking at the G band. Similarly, for flake position E, optical microscopy determined that the flake was positioned 21 % of the way across the channel. The position as determined from the Raman spectrum suggested the flake was displaced by around 25.8 % if looking at the D band or 23.8 % if looking at the G band. For flake position F, optical microscopy determined that the flake was positioned 45 % of the way across the channel. The position as determined from the Raman spectrum suggested the flake was displaced by around 48.9 % if looking at the D band or 49.6 % if looking at the G band. In all cases, the position predicted from both the D and G bands individually was within a few % of the optical microscopy measurement. It was estimated that the optical microscopy measurements had an error of approximately ±5 % due to the difficulty in accurately observing the flake edges and hence the centre of the flake.

Supplementary References

1. Dyakov, S. A. *et al.* Surface states in the optical spectra of two-dimensional photonic crystals with various surface terminations. *Phys. Rev. B* **86,** 115126 (2012).
2. Christ, A., Tikhodeev, S. G., Gippius, N. A., Kuhl, J. & Giessen, H. Waveguide-Plasmon Polaritons: Strong Coupling of Photonic and Electronic Resonances in a Metallic Photonic Crystal Slab. *Phys. Rev. Lett.* **91,** 183901 (2003).
3. Tikhodeev, S. G., Yablonskii, A. L., Muljarov, E. A., Gippius, N. A. & Ishihara, T. Quasiguided modes and optical properties of photonic crystal slabs. *Phys. Rev. B* **66,** 045102 (2002).
4. Dyakov, S. A. *et al.* Influence of the buffer layer properties on the intensity of Raman scattering of graphene. *J. Raman Spectrosc.* **44,** 803–809 (2013).
5. Dyakov, S. A. *et al.* Optical properties of grooved silicon microstructures: Theory and experiment. *J. Exp. Theor. Phys.* **113,** 80–85 (2011).
6. Ko, D. Y. K. & Inkson, J. C. Matrix method for tunneling in heterostructures: Resonant tunneling in multilayer systems. *Phys. Rev. B* **38,** 9945–9951 (1988).

7. Sengupta, A., Herminghaus, S. & Bahr, C. Liquid crystal microfluidics: surface, elastic and viscous interactions at microscales. *Liq. Cryst. Rev.* **2,** 73–110 (2014).

8. Li, J., Wen, C.-H., Gauza, S., Lu, R. & Wu, S.-T. Refractive Indices of Liquid Crystals for Display Applications. *J. Disp. Technol.* **1,** 51–61 (2005).

9. Hummers, W. S. & Offeman, R. E. Preparation of Graphitic Oxide. *J. Am. Chem. Soc.* **80,** 1339–1339 (1958).

10. Paredes, J. I., Villar-Rodil, S., Martínez-Alonso, A. & Tascón, J. M. D. Graphene Oxide Dispersions in Organic Solvents. *Langmuir* **24,** 10560–10564 (2008).

11. Tie, W. *et al.* Dynamic electro-optic response of graphene/graphitic flakes in nematic liquid crystals. *Opt. Express* **21,** 19867–79 (2013).

12. Shen, T.-Z., Hong, S.-H. & Song, J.-K. Electro-optical switching of graphene oxide liquid crystals with an extremely large Kerr coefficient. *Nat. Mater.* **13,** 394–9 (2014).

13. Twombly, C. W., Evans, J. S. & Smalyukh, I. I. Optical manipulation of self-aligned graphene flakes in liquid crystals. *Opt. Express* **21,** 1324–34 (2013).

Supplementary Video Captions

Supplementary video 1: Orientation switching of a graphene flake, dispersed in E7, in a 50 *μm* diameter microfluidic reservoir. A pulsed electrical field is applied and a corresponding change in the flake alignment and patterning of the LC surface are observed. The video was taken using cross-polarised light.

Supplementary video 2: Induced motion of GO flakes, dispersed in E7, in an 11.6 *μm* wide channel. A bias is applied across the channel and is steadily increased. Switching of the LC director occurs above a threshold applied field strength of 0.25 *V/μm*. As the LC director is switched, motion of the dispersed GO flakes is observed. The video was taken with cross-polarised light.

Supplementary video 3: Induced motion of a single GO flake, dispersed in MLC-6608, in an 11.6 *μm* wide channel. An electric field is applied across the channel and is increased and decreased periodically above and below the threshold applied field strength. Induced rotational and translational motion of the flake is observed. The video was taken using unpolarised light.

Supplementary video 4: Optically induced motion of GO flakes, dispersed in MLC-6608, in a 50 *μm* diameter microfluidic reservoir is observed. Motion can be seen as a change in the interference

pattern of the backscattered light from the Raman laser. Optical trapping with particles drawn to high light intensity regions is observed.
